# The use of ultrasensitive quantitative-PCR to assess the impact of primaquine on asymptomatic relapse of *Plasmodium vivax* infections: a randomized, controlled trial in Lao PDR

**DOI:** 10.1186/s12936-019-3091-5

**Published:** 2020-01-03

**Authors:** Koukeo Phommasone, Frank van Leth, Mallika Imwong, Gisela Henriques, Tiengkham Pongvongsa, Bipin Adhikari, Thomas J. Peto, Cholrawee Promnarate, Mehul Dorda, Pasathorn Sirithiranont, Mavuto Mukaka, Pimnara Peerawaranun, Nicholas P. J. Day, Frank Cobelens, Arjen M. Dondorp, Paul N. Newton, Nicholas J. White, Lorenz von Seidlein, Mayfong Mayxay

**Affiliations:** 1 Lao-Oxford-Mahosot Hospital-Wellcome Trust Research Unit (LOMWRU), Microbiology Laboratory, Mahosot Hospital, Vientiane, Lao PDR; 2 Department of Global Health, Amsterdam University Medical Centers, Location AMC, Amsterdam, The Netherlands; 3 Amsterdam Institute for Global Health & Development, Amsterdam, The Netherlands; 4 Mahidol Oxford Research Unit, Mahidol University, Bangkok, Thailand; 5 Department of Molecular Tropical Medicine and Genetics, Faculty of Tropical Medicine, Mahidol University, Bangkok, Thailand; 6 Savannakhet Provincial Health Department, Savannakhet, Savannakhet Province Lao PDR; 7 Centre for Tropical Medicine and Global Health, Nuffield Department of Medicine, University of Oxford, Oxford, UK; 8 WWARN Asia Regional Centre, Mahidol University, Bangkok, Thailand; 9 Institute of Research and Education Development, University of Health Sciences, Vientiane, Lao PDR

**Keywords:** Malaria, *P. vivax*, PCR, Primaquine, Relapse

## Abstract

**Background:**

Trials to assess the efficacy of the radical cure of *Plasmodium vivax* malaria with 8-aminoquinolines require that most post-treatment relapses are identified, but there is no consensus on the optimal duration of follow-up in either symptomatic or asymptomatic vivax malaria. The efficacy of a 14-day course of primaquine on the cumulative incidence of recurrent asymptomatic *P. vivax* infections detected by ultrasensitive quantitative PCR (uPCR) as a primary endpoint was assessed.

**Methods:**

A randomized, placebo-controlled, single-blind trial was conducted in four villages of the Lao PDR during 2016–2018 nested in a larger project evaluating mass drug administrations (MDA) with dihydroartemisinin-piperaquine (DP) and a single low-dose primaquine to clear *Plasmodium falciparum* infections. In the nested sub-study, eligible participants with mono- or mixed *P. vivax* infections detected by uPCR were randomized to receive either 14 days of primaquine (0.5 mg/kg/day) or placebo during the last round of MDA (round 3) through directly observed therapy. Participants were checked monthly for 12 months for parasitaemia using uPCR. The primary outcome was cumulative incidence of participants with at least one recurrent episode of *P. vivax* infection.

**Results:**

20 G6PD-normal participants were randomized in each arm. 5 (29%) of 20 participants in the placebo arm experienced asymptomatic, recurrent *P. vivax* infections, resulting in a cumulative incidence at month 12 of 29%. None of the 20 participants in the intervention arm had recurrent infections (p = 0.047 Fisher’s exact test). Participants with recurrent *P. vivax* infections were found to be parasitaemic for between one and five sequential monthly tests. The median time to recurrence of *P. vivax* parasitaemia was 178 days (range 62–243 days).

**Conclusions:**

A 14-day course of primaquine in addition to a DP-MDA was safe, well-tolerated, and prevented recurrent asymptomatic *P. vivax* infections. Long follow-up for up to 12 months is required to capture all recurrences following the treatment of asymptomatic vivax infection. To eliminate all malarias in settings where *P. vivax* is endemic, a full-course of an 8-aminoquinolines should be added to MDA to eliminate all malarias.

*Trial registration* This study was registered with ClinicalTrials.gov under NCT02802813 on 16th June 2016. https://clinicaltrials.gov/ct2/show/NCT02802813

## Background

*Plasmodium vivax* remains one of the major public health problems in malaria endemic countries where 2.5 billion people are at risk of infections [1]. The control of *P. vivax* has been slower than the control of *Plasmodium falciparum* due to its ability to lie dormant in liver cells (hypnozoites), causing relapse weeks to months after the initial attack. *Plasmodium vivax* gametocytes appear quite early, before the onset of clinical symptoms, resulting in mosquito infection and transmission. Moreover, low density *P. vivax* infections are missed by conventional diagnostic tests [2]. Another challenge in the control and eventual elimination of vivax malaria is the exact testing and treatment required to clear hypnozoites i.e. the radical cure. Primaquine and tafenoquine, both 8-aminoquinolines, are the only licensed drugs with activity against hypnozoites for radical treatment of *P. vivax* [3–5] but are under-utilized due to their potential to cause haemolysis in glucose-6-phosphate-dehydrogenase (G6PD) deficient people.

Trials to assess the efficacy of radical cure of *P. vivax* malaria with 8-aminoquinolines require prolonged follow up of a large sample of participants to detect clinically relevant reductions in the number of recurrent clinical *P. vivax* malaria episodes. Clinical as well as asymptomatic recurrences are epidemiologically important as they are the likely reservoir of the infection [6]. Indeed, since the application of PCR to malaria, asymptomatic *Plasmodium* carriers have been increasingly recognized as they are substantially more prevalent than clinical cases and probably serve as infective reservoirs [7, 8]. The recent development of a highly sensitive quantitative PCR (uPCR) to identify and quantify low-density *Plasmodium* infections by using a relative large blood volume, allows the reliable detection of parasite densities as low as 22 parasites/ml of blood [9]. In order to eliminate malaria, treatment of asymptomatic *P. vivax* carriers is critical to prevent the transmission of persistent *P. vivax* infections. Detection of asymptomatic parasitaemia by uPCR could be a critical trial endpoint in the assessment of the anti-relapse potential of anti-malarial drug regimens in asymptomatic infections. The objective of this study was to assess the efficacy of a 14-day radical cure with primaquine using the incidence of asymptomatic *P. vivax* infections detected by uPCR as endpoint.

## Methods

### Trial design

**Fig. 1:**
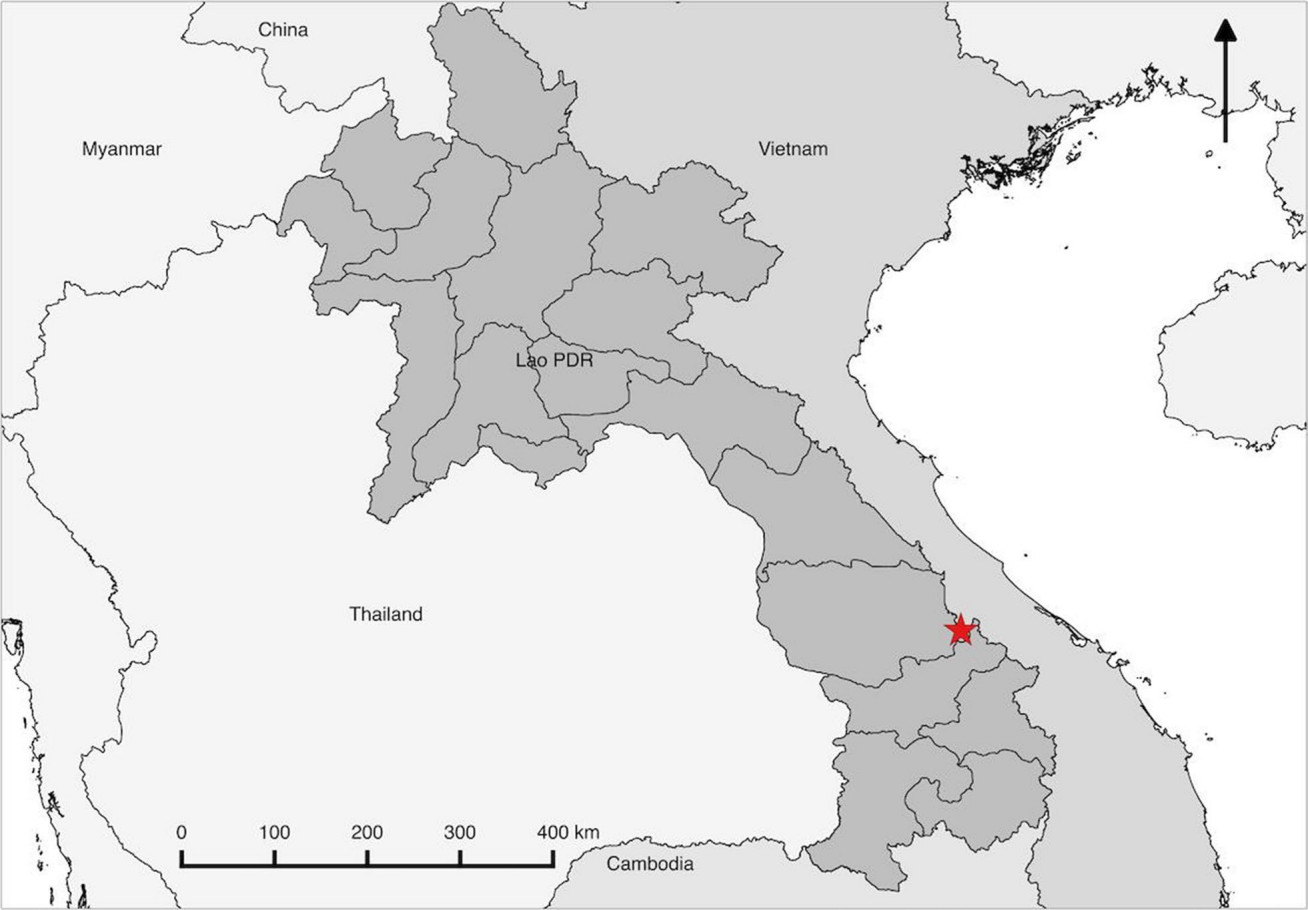
Map of study site (red star indicates study site)

This sub-study was nested within a large multicentre targeted malaria elimination project, a mass drug administration (MDA) trial in the Greater Mekong Subregion which included four villages of Nong District, Savannakhet Province, Lao PDR (Fig. 1) [10]. Two of the four villages were randomized to receive three rounds of MDA, each consisting of a 3-day course of dihydroartemisinin–piperaquine (DP) and a single low dose (0.25 mg/kg) of primaquine (SLDPQ). The other two villages served as controls and received MDA after 12 months of surveillance [10]. Participants in the MDA villages found to be infected with *P. vivax* by uPCR at the baseline survey or month 0 were invited to participate in the primaquine trial described here during MDA round 3. Participants in the control villages, who were found to be infected during cross-sectional surveys, were invited to participate during crossover MDA round 3 (month 14 of MDA trial). This sub-study was a nested, randomized, single-blind, treatment trial of asymptomatic vivax infections in participants without G6PD deficiency with asymptomatic *P. vivax* mono or mixed-infection detected during the MDA trial [11].

### Study site

The Lao PDR is a land-linked country in Southeast Asia bordered by China and Myanmar in the north, Vietnam in the east, Thailand in the west and Cambodia in the south. The country is composed of 18 provinces, which are further subdivided into 147 districts. Malaria epidemiology is highly heterogeneous; the five-southern provinces, including Savannakhet where the trial took place are the most malaria prevalent and accounted for 97% of cases reported in Lao PDR. *P. falciparum* is still the predominant parasite species, but *P. vivax* accounted for nearly 47% of reported cases in 2014. Lao PDR together with its neighbouring countries plans to eliminate malaria in the Greater Mekong Subregion by 2030 [12]. The national first-line treatment for *P. vivax* is a 3-day course of artemether-lumefantrine plus 14-day primaquine and the second-line treatment is 3-day chloroquine plus 14-day primaquine. A radical cure with primaquine policy was adopted by the national malaria control programme (CMPE) in 2011 but has not yet been widely implemented due to the lack of appropriate G6PD tests.

### Participants

Male, and non-pregnant and non-breastfeeding females older than 9 years were eligible to participate if asymptomatic *P. vivax* mono- or mixed infections were detected by ultrasensitive qPCR during the cross-sectional surveys preceding the MDAs. People with following conditions were excluded: unable to take oral treatment, previous episode of haemolysis or severe haemoglobinuria following primaquine, known hypersensitivity or allergic to study drugs, blood transfusion in the last 90 days, acute malaria episode requiring treatment or a febrile condition at the time of recruitment, anaemia with haemoglobin less than 9 g/dl. Participants who took medication that might interfere with pharmacokinetics of primaquine were also excluded. Participants were recruited at two different time points. The first recruitment was in the two intervention villages that received MDA at the beginning of the MDA trial. The second recruitment was 12 months later in the two control villages when participants received the cross-over MDA at the end of the surveillance period. All study participants had received 3 rounds consisting each of three doses DP + SLDPQ except for two participants who received two rounds of DP + SLDPQ. A single round DP + SLDPQ is sufficient to clear *P. vivax* blood stages (schizontocides) and a SLDPQ given has no effect on the hypnozoites of *P. vivax*.

### Intervention

Participants who met the inclusion criteria including informed consent were randomly assigned to 14 days of primaquine (0.5 mg/kg for 14 days) or placebo in addition to the 3 day-course of dihydroartemisinin-piperaquine (7 mg/kg/day DHA and 55 mg/kg/day piperaquine) they had received during MDA. Day 0 for the current sub-study corresponded to month 2 of the MDA trial (third MDA round) in the intervention villages or month 14 in the control villages which was also the third round of the MDA. DP used in our study was manufactured by Guilin Pharmaceutical Company, China. Primaquine and placebo were manufactured by the Government Pharmaceutical Organization, Thailand, and had a similar appearance.

### Outcomes

The primary outcome was the cumulative incidence of asymptomatic *P. vivax* recurrences detected by uPCR over 12-month follow-up. Secondary outcomes were parasite density, time to first recurrence, frequency of recurrent asymptomatic and clinical malaria episodes, changes in haemoglobin (Hb) concentration, and the number of adverse events over the first 28 days (until 14 days after the last dose of primaquine or placebo). Time to parasite clearance could not be assessed in this trial.

### Sample size

The sample size was chosen for mainly pragmatic reasons with the aim to enrol up to 60 participants. Prior to study start neither the asymptomatic *P. vivax* prevalence in Savannakhet nor the impact of primaquine on asymptomatic *P. vivax* infections was known. Assuming that relapse would be detectable by uPCR in 30% of the participants in the control arm a sample size of 60 participants, 30 per arm, would be sufficient to detect that difference between groups in clearing parasitaemias based on alpha value of 0.05, a power of 80%, and 20% loss to follow-up.

### Randomization

The computer-generated randomization list was prepared centrally at the Mahidol-Oxford Tropical Medicine Research Unit (MORU) with a group ratio of 1:1. Regimen allocation was kept in a series of sealed, opaque envelopes that were sequenced numerically. Participants were sequentially assigned to the envelopes, which contained the random treatment allocation.

### Blinding

The treatment allocation was concealed to participants and laboratory technicians who performed uPCR throughout the study.

### Procedures

On day 0, a physical examination was conducted, socio-demographic data, a history of illness and medication in the last 28 days was collected and 3 ml of blood was taken for haemoglobin measurement and for uPCR before taking study drugs. Directly observed therapy (DOT) was used to ensure adherence. Drugs were administered with biscuits and soya milk to reduce gastro-intestinal side effects. After drug administration, participants were observed for an hour. If a participant vomited within 30 min, the full dose was repeated. If the participant vomited after 30 min but less than 1 h, half of the dose was given. Temperature and adverse events were collected on daily basis over the first 14 days and then on day 28. All adverse events either related or unrelated to study drugs during this period were recorded. If hospitalization, death, or a drop in haemoglobin by 25% compared to baseline occurred, it was to be recorded as serious adverse event. Follow-up blood samples were taken on Day 2, 6, 13, 28 and then monthly over 1 year for uPCR and haemoglobin measurement. During the follow-up visits temperature and history of illness during the preceding month were recorded. Data from each participant were recorded in a standardized case record form. Participants found to have recurrent infections detected by uPCR during follow-up period without clinical symptoms were not treated. Participants with clinical signs and symptoms of malaria and positive for *Plasmodium* infection by rapid diagnostic test were treated according to Lao national malaria treatment guidelines.

## Laboratory procedures

### Sample collection

A 3 ml blood sample was collected into an EDTA anticoagulated tube, kept in an ice packed cool box and transported within 6 h from the villages to the local laboratory. On arrival at the laboratory, 200 microlitre samples were aliquoted for haemoglobin measurement, and the remaining blood was processed and separated into red blood cell pellets, buffy coat and plasma. Each aliquot was stored at − 20 °C in a freezer along with an additional negative control in the sample pool. The samples were transported on dry ice to the molecular laboratory of the MORU in Bangkok, Thailand for uPCR analysis.

### DNA extraction and PCR amplification

A highly sensitive and specific high-volume quantitative PCR method was used, which has a lower limit of detection of 22 parasites/ml [9]. In short, an automated DNA extraction method (QIAsymphony and DSP DNA midi kit; Quiagen, Germany) was used to purify DNA from thawed red blood cells. The purified DNA was concentrated, dried and then used as a template for PCR detection and quantification of Plasmodium. DNA of Plasmodium was detected and quantified using 18S rRNA-targeting primers and hydrolysis probes. For Plasmodium-positive samples, an attempt was made to identify the species using *P. falciparum* and *P. vivax*-specific PCR primers [9].

### Other field laboratory work

Haemoglobin levels were measured in the field by using the HemoCue^®^ Hb 301 system (Hemocue AB, Angelholm, Sweden) by trained laboratory technician following the manufacturer’s recommendation. G6PD deficiency was tested by using the fluorescent spot test (FST) (Trinity Biotech Plc, IDA Business Park, Bray, Co Wicklow, Ireland), which showed a perfect match with spectrophotometry at 30% cut-off activity [13].

### Statistical analysis

All data collection was transferred into databases for data management and cleaning using Macro electronic data capture. An intention-to-treat (ITT) analysis was performed to determine primary and secondary outcomes, with the ITT defined as all randomized participants who took at least one dose of primaquine. The cumulative incidence of *P. vivax* infections over 12-month follow-up was assessed by survival analysis. Follow-up data were censored for participants without events throughout the follow-up period, and right censored at the day of their first recurrence or the day when they were last seen which ever came first. The difference between the two survival curves was assessed through Kaplan–Meier estimates at month 12 using the log-rank test. Time to first recurrence was calculated as time from the start of the intervention (D0 of administering the 14-day primaquine regimen) to time when a follow-up sample turned positive and displayed in number of days and range. Given the small sample size and the small number of outcome events, we did not perform any other analyses (e.g. Cox regression), as adequate inference of statistical models in this situation is not possible. To count the total number of recurrent *P. vivax* episodes per person through the available follow-up, there was no censoring to include multiple episodes. The effect of primaquine on haemoglobin levels was assessed by using a multilevel mixed effect linear model with an unstructured covariance to accommodate their repeated measurements. Adverse events were reported by frequency. Statistical significance was assumed at the 5% level. The analysis was performed using Stata version 14.1 (StataCorp, Texas, USA).

## Results

The first 18 participants were enrolled in June 2016 and another 22 participants in June 2017. The last follow-up visit was on 15th June 2018. In total, 40 participants were randomized (20 in each arm). The baseline characteristics of trial participants were balanced between the treatment arms (Table 1). 16 (80%) participants in each arm completed the 12 month-follow-up period. Four participants in each arm did not complete 12-month follow-up.

**Table 1:** Participant characteristics at baseline

Variable	Drug allocation		Total, N = 40
	Placebo, n = 20	Primaquine, n = 20	
Age in year, mean (95% CI)	31.7 (24.4–39.0)	23.4 (17.1–29.6)	27.5 (22.6–32.4)
Male (%)	14 (70)	12 (60)	26 (65)
Weight kg, mean (95% CI)	44.5 (39.7–49.3)	44.6 (38.2–50.9)	44.5 (40.6–48.5)
Complete 12-month FU (%)	16 (80)	16 (80)	32 (80)
Hb^a^ g/dl, mean (95% CI)	13.7 (13.1–14.4)	13.5 (12.9–14.1)	13.6 (13.2–14.1)
Age in year, median (Range)	32.5 (10–76)	19 (10–54)	25.5 (10–76)
Weight kg, mean (95% CI)	44.5 (39.7–49.3)	44.6 (38.2–50.9)	44.5 (40.6–48.5)
Recruitment year, n			
Jun-16	9	9	18
Jun-17	11	11	22
uPCR			
During surveys of MDA trial			
Mono-PV infection	16	17	33
Mixed PV infection	4	3	7
At day 0			
PV infection	0	0	0

^a^Haemoglobin

**Fig. 2:**
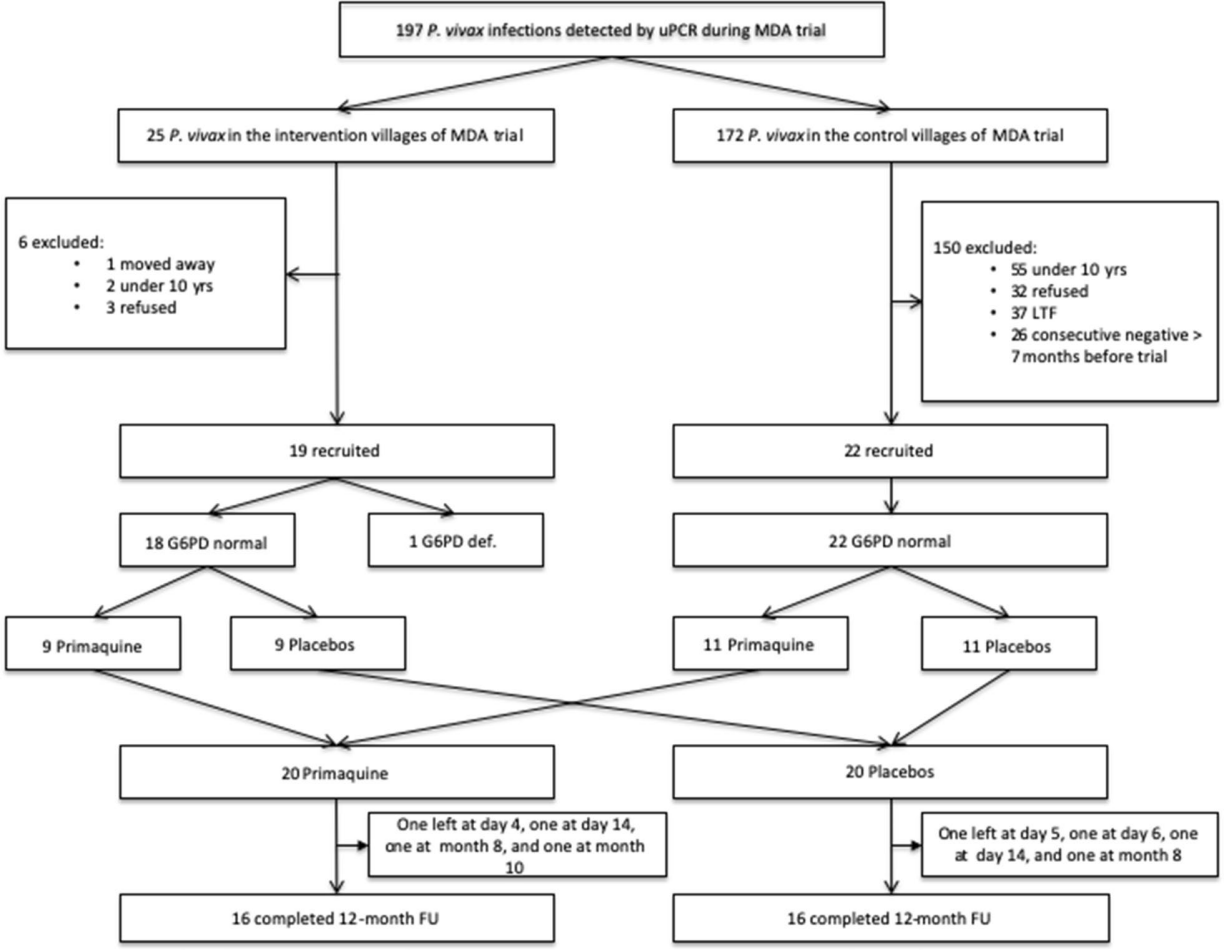
Consort flow chart of recruitment. *uPCR* ultrasensitive polymerase chain reaction, *G6PD* glucose 6 phosphate dehydrogenase deficiency, *FU* follow-up

Discontinuations occurred in the intervention arm on day 4, day 14, month 8 and 10, and in the control arm on day 5, day 6, day 14 and 8 (Fig. 2). The reason for leaving the study by the first 14 days, was “enough of frequent blood draws”, while the participants who left by Month 8 or month 10 were lost to follow-up. The primaquine treatment was given at the start of DP during the MDA round 3 with the median dosing of 0.52 mg/kg per day (range 0.35 to 0.77 mg/kg). During the 12-month follow-up period, none of participants developed clinical *P. vivax* infection, but one participant in placebo arm developed clinical *P. falciparum* at month 5 and was treated with 3-day course of artemether–lumefantrine according to Lao national malaria treatment guidelines and recovered well.

### *Plasmodium vivax* recurrent infections

**Fig. 3:**
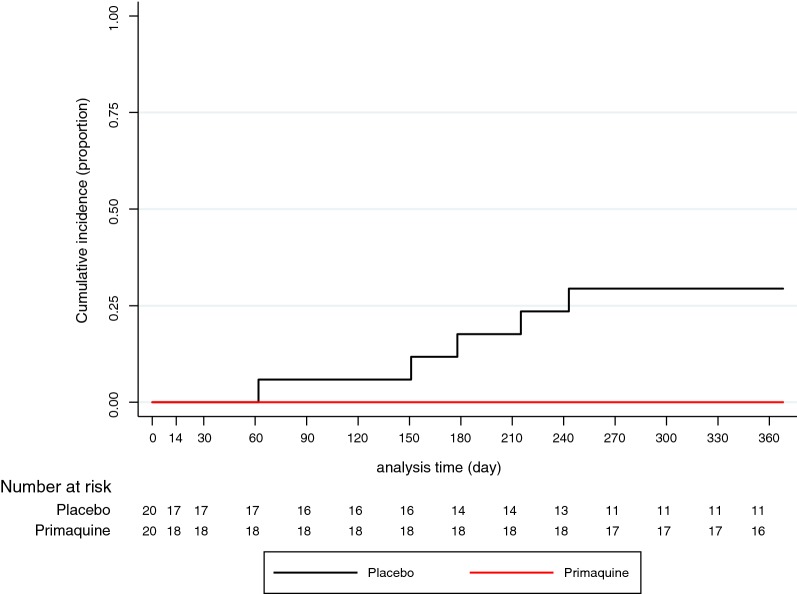
Cumulative recurrent incidence of *P. vivax* infections by intervention

**Fig. 4:**
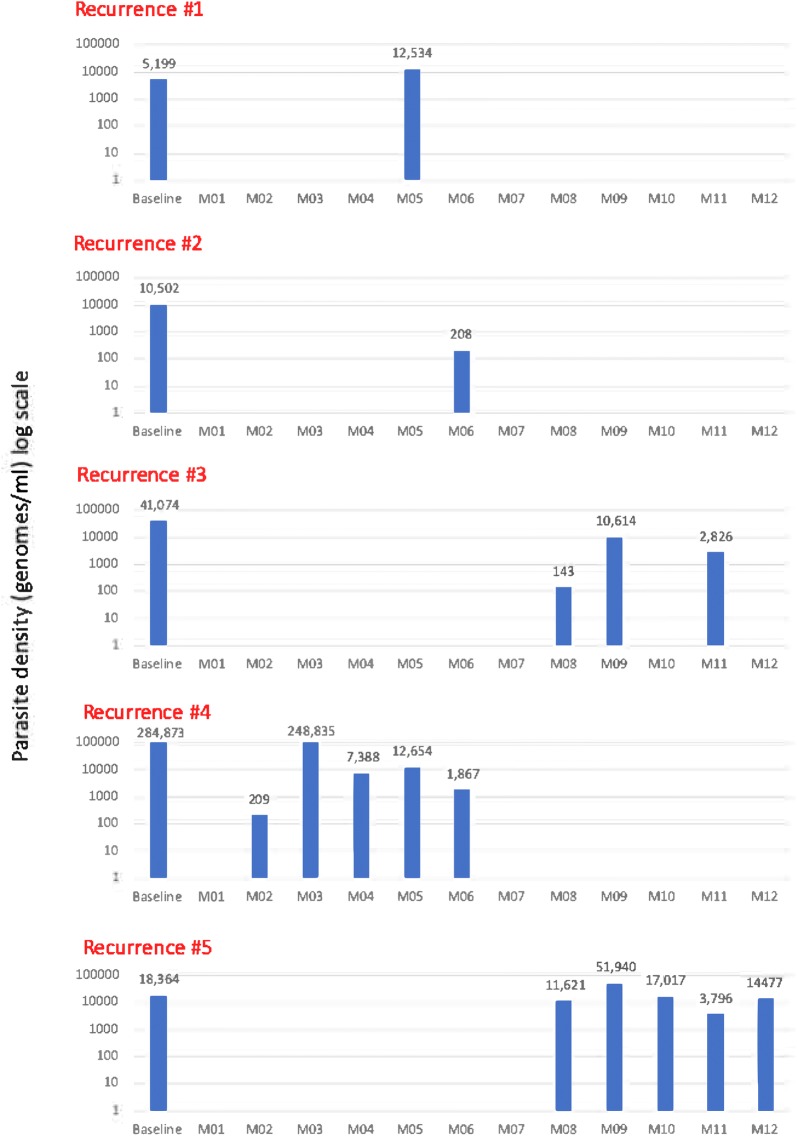
The pattern of recurrent *P. vivax* infections in 5 study participants all in the placebo group. The x-axis shows the time of the survey in relation to the drug administration (D = Day, M = Month; Baseline of Recurrent 1, 2, and 3 = M0 of malaria elimination project; Baseline of Recurrent 4 and 5 = During cross sectional surveys of malaria elimination project either M6, M9 or M12). The y-axis shows the density (genomes/ml) on a log scale. The numbers above the columns indicate the parasite density at that point in time

Five participants had at least one recurrent *P. vivax* infection in the placebo arm, resulting in a cumulative incidence at month 12 of 29% [95% confidence interval (CI) 13.4–56.9], and none in the primaquine arm (p = 0.047 Fisher’s exact test) (Fig. 3). The median time to first recurrence in the placebo arm was 178 days (range 62–243 days). The pattern of recurrent infections was variable (Fig. 4). Participants with recurrent *P. vivax* infections were found to be parasitaemic between one and five sequential monthly tests. The participant with the highest parasite density at M0 (Recurrence #4; 284,873 genomes/ml) had no apparent lag between the first and five subsequent tests. The participant with the lowest parasite density (Recurrence #1; 5190 genomes/ml) tested only once positive at M06 (6 months after the start of the trial). No clinical *P. vivax* cases were detected during the follow-up period.

### Effect of primaquine on haemoglobin level over the first 28 days

**Fig. 5:**
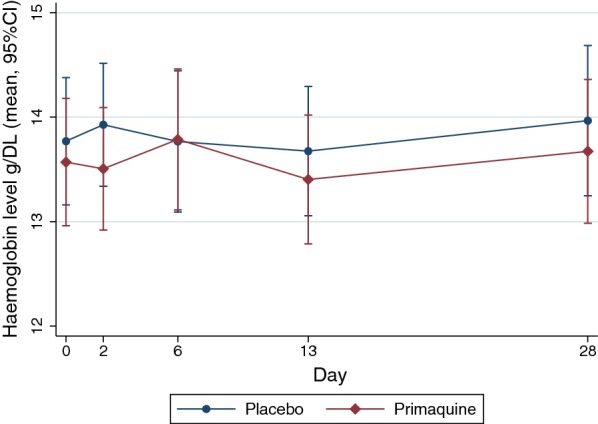
Changes in haemoglobin level of participants in primaquine and placebo arms over the first 28 days after drug administration

A small decrease in haemoglobin level of 0.225 g/dl and 0.080 g/dl was recorded on day 2 and day 13, respectively in the primaquine group but was not clinically significant (Fig. 5). A multilevel mixed effect model to assess the effect of drug and time on haemoglobin level showed that the mean haemoglobin level of participants who took primaquine was 0.228 g/dl (95% CI − 1.058 to 0.602) lower than those who took the placebo (p-value: 0.59).

### Adverse events

Two participants reported adverse events in the primaquine arm which were considered related to study drugs; one participant felt dizzy, while the other felt dizzy and nauseated 30 min after taking study drugs. Both adverse events were mild and self-limited. Two adverse events were detected over the first 28 day-follow-up in the placebo arm, one participant reported watery stool, which was considered as possibly related to the study drug, while the other, a foot injury was considered unrelated. No patient complained of red or black urine and no serious adverse events were reported.

## Discussion

In this placebo-controlled evaluation, nested within the study of dihydroartemisinin-piperaquine mass antimalarial drug treatment conducted in Lao PDR, a 14-day primaquine regimen of 0.5 mg/kg/day following a three-day course of DP was well tolerated and effective in the prevention of recurrences over a period of 12-month follow-up in participants with asymptomatic *P. vivax* infection. None of the participants in primaquine arm had recurrent *P. vivax* infections. The primaquine dose used in our study was double the standard dose recommended by the Lao national malaria treatment guideline. However, the World Health Organization, US Centre for Disease Control and many European countries have recommended this higher dose for *P. vivax* infections in East Asia and Oceania [14]. Provided patients with G6PD deficiency are excluded, this dose has been shown to be safe [15, 16]. Although safety in G6PD heterozygotes not identified by the fluorescent spot test remains an open question [17]. In this small study, the higher primaquine dose was safe and well tolerated by our participants without a clinically relevant drop in haemoglobin levels. The administration of at least one full course of schizontocidal drugs, DP with a SLDPQ without a full course of an 8-aminoquinoline, had no apparent impact on recurrent vivax infections. The study highlights once more the critical need for the radical cure with an 8-aminoquinoline to eliminate all malarias in vivax endemic regions [18]. The radical treatment of *P. vivax* can consist of 14-day primaquine or a single dose of tafenoquine. To adhere to 14 days of primaquine for successful treatment is important but can be challenging. As a consequence, many trials have tried to shorten the regimen varying the cumulative dose of primaquine and the duration of treatment. 7-day high dose primaquine (total dose of 7 mg/kg) is as efficacious as standard 14-day high-dose primaquine in radical treatment of vivax malaria at 1 year follow-up, but quantitative G6PD testing is required as there is a higher risk of haemolysis in treatments with a higher daily primaquine dose [15, 16]. However, treatment for less than 7 days has proved to be less effective [19]. Takeuchi et al. compared DOT for 14 days of primaquine versus non- DOT primaquine, and found that the Non-DOT group experienced more recurrences [20]. Novel, robust, quantitative G6PD tests are already available and more products are under development [21, 22]. The combination of reliable G6PD testing combined with safe and effective 8-aminoquinoline regimes holds promise for the elimination of all malarias.

Recurrent asymptomatic *P. vivax* infections were seen throughout the follow-up period but only in the control group which had not received primaquine. Recurrent infections can have three possible causes. They can be caused by recrudescent or persistent infections which is unlikely considering the delay between schizonticidal treatment and observed infection. They can also be due to re-infections caused by a new mosquito bite. This explanation is not likely in this study as no new infections were observed in the participants who had received a full course primaquine which clears all hypnozoites. Within 1 month of primaquine treatment the participants in the primaquine group had the same risk of becoming re-infected as in the placebo group but had no infections. This observation suggests that the *P. vivax* transmission in the study site is low and the recurrent infections in the control group are most likely due to relapses due to the activation of hypnozoites.

This study shows the potential of using uPCR as a tool to assess the primary endpoint of recurrent infections without obvious clinical outcomes. Participants with recurrent infections showed no clinical signs related to their *P. vivax* infections which are likely to include gametocytes at some point in time and hence continue to contribute to the transmission of *P. vivax* [2, 23]. It is of note that the participant with the highest parasite load at enrolment had the shortest lag time to the first recurrence and was found to be infected with *P. vivax* during the following 5 surveys. By contrast, the participant with the lowest parasite density at enrolment had the first documented recurrent infection 6 months after enrolment. This observation would support the notion of a parasite density related recurrence rate. However, this is speculative as the number of participants with a recurrent infection is very low in this study. The South East Asian region is known to have a short lag time to the first recurrent infection which is on average 41 days [1, 24]. Short interval relapses are usually caused by tropical *P. vivax* strain, while temperate and sub-tropical strains have long incubation periods for relapse [25]. Time from initial infection to relapse and relapse frequency are not only determined by geographic origin of *P. vivax* strains but also number of inoculated sporozoites received from infected mosquitoes. The more sporozoites the liver harbours, the greater the chance to get illness early and the greater the frequency of relapses [26].

For comparison with historical studies, the outcome of this study can be rephrased as an incidence rate in the placebo group of 35.6 recurrent episodes/100 person-years (95% CI 14.8 to 85.5). This rate of recurrent asymptomatic infections detected by uPCR can be compared with the recurrence rate observed following clinical vivax malaria episodes in historical studies. In a recent large vivax malaria treatment trial [16] the recurrence rate over a year was 48.7 recurrent episodes/100 person-years (95% CI 43.4–54.4). As relapses following treatment of asymptomatic infections appear to have less periodicity (and thus early clustering) than those which follow symptomatic infections, long follow-up is required. It is, therefore, unlikely that treatment trials in people with subclinical infections can curtail the study period required for trials. Trialists may still be interested in recruiting people with subclinical infections to study radical curative treatments, as the prevalence of subclinical infections is much higher than of clinical episodes. None of the *P. vivax* infections detected in this study had any clinical signs or symptoms of malaria.

The study has several limitations. The sample size was very small; the number of people with *P. vivax* infections was lower than expected, limiting our enrolment to 40 participants. Secondly, the study was conducted following 3 rounds of MDA. While the first 18 participants (in the intervention villages of MDA trial) were recruited and randomized for trial within 2 months of the first positive uPCR, the remaining 22 participants were recruited in control villages 12 months later. Despite this, the distribution of participants with recurrent infections was similar in these two periods of time. There were no differences in climate between the 2 years, something that could have influenced the risk for reinfection. Thirdly, uPCR is a sophisticated tool that cannot be used in the field leading to a delay between blood collection and uPCR result. Lastly, more frequent follow-up of blood draws with uPCR might give insight into persistence of the infection.

## Conclusions

In the context of mass drug administration and appropriate G6PD testing, a 0.5 mg/kg/day dose of primaquine for 14 days following three rounds of dihydroartemisinin-piperaquine was safe, well-tolerated and effective in the prevention of recurrence of asymptomatic *P. vivax* infections. The elimination of all malarias could be much accelerated by the roll out of the radical cure with high dose primaquine or tafenoquine.

## Data Availability

The data are available upon request to the Mahidol Oxford Tropical Medicine Research Unit Data Access Committee (http://www.tropmedres.ac/data-sharing) for researchers and following the Mahidol Oxford Tropical Medicine Research Unit data access policy (http://www.tropmedres.ac/_asset/file/datasharing-policy-v1-1.pdf). Queries and applications for datasets should be directed to Rita Chanviriyavuth (rita@tropmedes.ac).

## Abbreviations

95% CI: 95% confidence interval
CMPE: Lao National Malaria Control Programme
D0: day zero
dL: decilitre
DOT: directly observed therapy
DP: dihydroartemisinin–piperaquine
EDTA: ethylenediaminetetraacetic acid
FST: fluorescent spot test
g: gram
G6PD: glucose 6 phosphate dehydrogenase
Hb: haemoglobin
ITT: intention-to-treat
kg: kilogram
Lao PDR: Lao People’s Democratic Republic
M0: month zero
MDA: Mass Drug Administration
mg: milligram
ml: millilitre
MORU: Mahidol-Oxford Research Unit
°C: degrees celsius
rRNA: ribosomal ribonucleic acid
SLDPQ: single low dose primaquine (0.25 mg/kg)
uPCR: ultrasensitive quantitative polymerase chain reaction
US: United States
