# Correction to: The use of ultrasensitive quantitative-PCR to assess the impact of primaquine on asymptomatic relapse of *Plasmodium vivax* infections: a randomized, controlled trial in Lao PDR

**DOI:** 10.1186/s12936-020-3124-0

**Published:** 2020-01-21

**Authors:** Koukeo Phommasone, Frank van Leth, Mallika Imwong, Gisela Henriques, Tiengkham Pongvongsa, Bipin Adhikari, Thomas J. Peto, Cholrawee Promnarate, Mehul Dhorda, Pasathorn Sirithiranont, Mavuto Mukaka, Pimnara Peerawaranun, Nicholas P. J. Day, Frank Cobelens, Arjen M. Dondorp, Paul N. Newton, Nicholas J. White, Lorenz von Seidlein, Mayfong Mayxay

**Affiliations:** 1 Lao-Oxford-Mahosot Hospital-Wellcome Trust Research Unit (LOMWRU), Microbiology Laboratory, Mahosot Hospital, Vientiane, Lao PDR; 2 Department of Global Health, Amsterdam University Medical Centers, Location AMC, Amsterdam, The Netherlands; 3 Amsterdam Institute for Global Health & Development, Amsterdam, The Netherlands; 4 Mahidol Oxford Research Unit, Mahidol University, Bangkok, Thailand; 5 Department of Molecular Tropical Medicine and Genetics, Faculty of Tropical Medicine, Mahidol University, Bangkok, Thailand; 6 Savannakhet Provincial Health Department, Savannakhet, Savannakhet Province Lao PDR; 7 Centre for Tropical Medicine and Global Health, Nuffield Department of Medicine, University of Oxford, Oxford, UK; 8 WWARN Asia Regional Centre, Mahidol University, Bangkok, Thailand; 9 Institute of Research and Education Development, University of Health Sciences, Vientiane, Lao PDR

## Correction to: Malar J (2020) 19:4 10.1186/s12936-019-3091-5

Following publication of the original article [1], it was brought to the authors’ attention that one of the names in the author list had been provided with the incorrect spelling.

Namely, ‘Mehul Dhorda’ had been incorrectly spelled as ‘Mehul Dorda’.

The error has since been corrected in the original article.

The authors apologize for any inconvenience caused.
